# An integrated approach identifies IFN-regulated microRNAs and targeted mRNAs modulated by different HCV replicon clones

**DOI:** 10.1186/1471-2164-12-485

**Published:** 2011-10-04

**Authors:** Roberto Bruni, Cinzia Marcantonio, Elena Tritarelli, Paola Tataseo, Emilia Stellacci, Angela Costantino, Umbertina Villano, Angela Battistini, Anna Rita Ciccaglione

**Affiliations:** 1 Department of Infectious, Parasitic and Immunomediated Diseases, Istituto Superiore di Sanità, Rome, Italy; 2 ASL Avezzano-Sulmona, Transfusional Medicine and Molecular Biology Laboratory, Sulmona, Italy; 3 Department of Hematology, Oncology and MolecularMedicine, Istituto Superiore di Sanità, Rome, Italy

## Abstract

**Background:**

Infections with hepatitis C virus (HCV) progress to chronic phase in 80% of patients. To date, the effect produced by HCV on the expression of microRNAs (miRs) involved in the interferon-β (IFN-β) antiviral pathway has not been explored in details. Thus, we compared the expression profile of 24 selected miRs in IFN-β-treated Huh-7 cells and in three different clones of Huh-7 cells carrying a self-replicating HCV RNA which express all viral proteins (HCV replicon system).

**Methods:**

The expression profile of 24 selected miRs in IFN-β-treated Huh-7 cells and in HCV replicon 21-5 clone with respect to Huh-7 parental cells was analysed by real-time PCR. To exclude clone specific variations, the level of 16 out of 24 miRs, found to be modulated in 21-5 clone, was evaluated in two other HCV replicon clones, 22-6 and 21-7. Prediction of target genes of 3 miRs, confirmed in all HCV clones, was performed by means of miRGator program. The gene dataset obtained from microarray analysis of HCV clones was farther used to validate target prediction.

**Results:**

The expression profile revealed that 16 out of 24 miRs were modulated in HCV replicon clone 21-5. Analysis in HCV replicon clones 22-6 and 21-7 indicated that 3 out of 16 miRs, (miR-128a, miR-196a and miR-142-3p) were modulated in a concerted fashion in all three HCV clones. Microarray analysis revealed that 37 out of 1981 genes, predicted targets of the 3 miRs, showed an inverse expression relationship with the corresponding miR in HCV clones, as expected for true targets. Classification of the 37 genes by Panther System indicated that the dataset contains genes involved in biological processes that sustain HCV replication and/or in pathways potentially implicated in the control of antiviral response by HCV infection.

**Conclusions:**

The present findings reveal that 3 IFN-β-regulated miRs and 37 genes, which are likely their functional targets, were commonly modulated by HCV in three replicon clones. The future use of miR inhibitors or mimics and/or siRNAs might be useful for the development of diagnostic and therapeutic strategies aimed at the recovering of protective innate responses in HCV infections.

## Background

Infection with hepatitis C virus (HCV) represents the major cause of liver disease, affecting more than 170 million individuals worldwide. After a sub-clinical phase, greater than 80% of patients progress to persistent HCV infection, the leading cause of chronic liver disease associated with cirrhosis and hepatocellular carcinoma [1,2].

In the last years, microarray technology provided a comprehensive analysis of alterations in gene expression induced by HCV and revealed important processes of virus-host interactions [3-7]. Interestingly, microarray studies indicated that HCV stimulates the endogenous Type I Interferon (IFN-α/β) pathway as suggested by activation of IFN-stimulated genes (ISGs) [8-15]. Recently, it has been proposed that also microRNAs (miRs), a class of small non-coding regulatory RNAs, are involved in the antiviral pathway induced by IFN-β treatment. The synthetic introduction of five IFN-β-induced miRs into HCV replicon cells may simulate the antiviral effect of IFN-β blocking HCV replication and infection. These five miRs (miR-196, miR-296, miR-351, miR-431 and miR-448) likely induced an antiviral state either through alteration of gene expression and/or directly targeting HCV RNA, as was demonstrated for two of them (miR-196 and miR-448) [16].

Although HCV activates the endogenous IFN-α/β pathway it conversely shows an impressive ability to induce persistent infections. Indeed, it is also clear that HCV has evolved several mechanisms to control the IFN antiviral response, inhibiting the pathway at different levels [17]. Recently, it has been suggested that an improper pre-activation of ISGs in the liver of HCV infected patients may hinder the antiviral response. The discovery of a genetic polymorphism in the interleukin 28B (IL28B) region on chromosome 19 of HCV patients depicted a more complex virus-host interaction. The IL28B non-CC variant has been associated with non-response to the IFN therapy and with lower rates of spontaneous clearance of HCV infection. The poor-response variant is also associated with higher intrahepatic expression level of ISGs [18-20].

A missing aspect in this scenario is the study of the effect produced by HCV on the expression of IFN-β-induced miRs. This is a relevant issue to understand how the virus can suppress the innate antiviral signaling and induce a persistent infection.

In a previous paper, we identified a common transcriptional response of Huh-7 cells to different clones of full-length HCV replicon [21]. Although a more advanced HCV cell culture models that release HCV viral particles has been developed [22-24], the replicon system has the advantage of taking into account the cellular gene expression variability of different HCV replicon cell clones. This approach allows searching for modulated genes shared by all clones, which are likely to be strictly needed for viral replication in different cellular contexts.

On this basis, we used the replicon system to identify IFN-regulated miRs that are modulated by HCV RNA replication. In particular, we analyzed the expression profile of 24 selected miRs in IFN-β-treated Huh-7 cell line and in three cell clones carrying a full-length HCV replicon (clones 21-5, 22-6 and 21-7). Among the identified 16 miRs modulated in the 21-5 clone, 3 miRs showed concordant expression when analyzed in the two other HCV replicons. By a combined approach, based on bioinformatic prediction and microarray analysis, we also identified 37 genes, targeted by the 3 miRs, which are involved in pathways and biological processes potentially implicated in the control of antiviral response by HCV infection.

## Results

### Expression of IFN-β-regulated miRs in 21-5 HCV replicon cells and in IFN-β-treated Huh-7 cells

To determine the impact of HCV RNA replication and protein synthesis on IFN-β-regulated miRs, we compared the expression profile of selected miRs in 21-5 cells, harbouring a full-length HCV genome, and in IFN-β-treated Huh-7 cells with the Huh-7 parental cell line.

In particular, the list of assayed miRs includes (a) eight IFN-β-induced miRs, which displayed complementarity in their seed sequences with HCV RNA genome (miR-1, miR-30, miR-128, miR-196, miR-296, miR-351, miR-431, miR-448) [16], (b) two miRs reported as IFN-β-unresponsive miRs (miR-125 and miR-142) [16], (c) miR-122a that promotes HCV RNA replication [25] and (d) three miRs (miR-155, miR-146a and miR-146b) modulated in innate immune response in monocytes/macrophages [26,27]. Actually, the name of five of the above miRs (miR-30, miR-128, miR-196, miR-125 and miR-142) indicates miR families, not just individual mature miR species: thus, we analysed the level of each member of those families. Overall, the expression profile of 24 miRs in 21-5 and IFN-β-treated Huh-7 cell lines was analysed (Table 1). Three miRs (miR-351, miR-431 and miR-448) showed an expression level below the detection limit of the assay (Ct ≥40), while five miRs were not differentially expressed in 21-5 cells (miR-125b, miR-30e-5p, miR-30e-3p, miR-30a-5p, miR-30d: fold-change values < 1.2 or > -1.2 in 21-5 cells). These eight miRs were not evaluated further.

**Table 1:** Fold-change of 24 selected miRs in IFNβ-treated Huh-7 and 21-5 cell lines vs. Huh-7

	Huh-7+ IFNβ	21-5
miR-1	**30, 13**	**22, 11**
miR-30a-3p	**4, 46**	**8, 79**
miR-146a	**1, 54**	**2, 73**
miR-142-5p	**2, 83**	**2, 20**
miR-146b	**2, 09**	**2, 11**
miR-142-3p	**2, 09**	**1, 74**
miR-296	**-1, 47**	**-1, 40**
miR-155	**-1, 69**	**-1, 61**
miR-128a	**-1, 47**	**-1, 88**
miR-128b	**-3, 30**	**2, 09**
miR-196a	**1, 31**	**-1, 50**
miR-125a	1, 05	**3, 43**
miR-196b	1, 11	**2, 92**
miR-30c	-1, 12	**1, 30**
miR-30b	-1, 04	**1, 28**
miR-122a	1, 14	**-1, 63**
miR-125b	-1, 10	1, 19
miR-30e-5p	1, 14	1, 18
miR-30e-3p	**-2, 12**	1, 17
miR-30a-5p	**-1, 38**	1, 15
miR-30d	-1, 12	1, 02
miR-351	n.d.	n.d.
miR-431	n.d.	n.d.
miR-448	n.d.	n.d.

In bold: fold-change values > 1.2 or < -1.2

n.d.: not detected, i.e. miRs showing an expression level below the detection limit of the assay (Ct ≥ 40)

**Figure 1:**
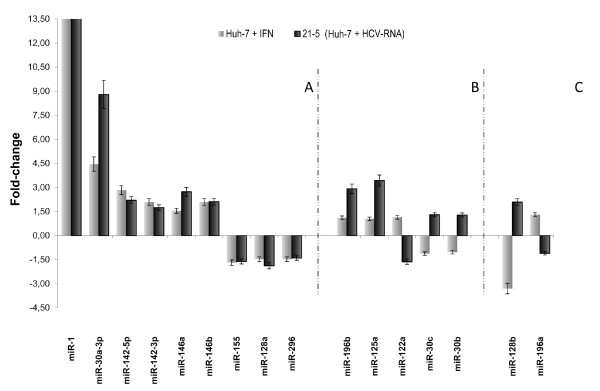
**Fold-change of 16 miRs modulated in IFN-β-treated Huh-7 cells and in 21-5 replicon cells**. Total RNA from both cell lines was used to assess miR levels using real time RT-PCR. miR levels from Huh-7 cells were set as the basis for the comparative results. miRs with a fold-change > 1.2 or < -1.2 were considered differentially expressed in 21-5 *vs*. Huh-7 cells. The mean of three independent biological replicas ± SD is reported; the RNA extract from each biological replica had been assayed by real-time RT-PCR in triplicate (three technical replicas per each RNA extract). (A) Concordant modulation (up- or down-regulation) of 9 miRs (miR-1, miR-30a-3p, miR-142-5p, miR-142-3p, miR-146a, miR-146b, miR-155, miR-128a and miR-296) in IFN-β-treated Huh-7 cells and in 21-5 replicon cells. (B) Modulation of five miRs (miR-196b, miR-125a, miR-122a, miR-30c and miR-30b) in 21-5 replicon cells. (C) Discordant modulation of two miRs (miR-128b and miR-196a) in IFN-β-treated Huh-7 cells and in 21-5 replicon cells.

The expression profile of the remaining 16 miRs revealed that they were modulated by IFN-β and/or HCV (Figure 1). In particular, concordant modulation (up- or down-regulation) of 9 miRs (miR-1, miR-30a-3p, miR-142-5p, miR-142-3p, miR-146a, miR-146b, miR-155, miR-128a and miR-296) was observed in IFN-β-treated Huh-7 cells and in 21-5 replicon cells (Figure 1, panel A). This result indicates that HCV replication can induce a miR signature as IFN-β treatment. Five miRs were modulated in 21-5 replicon cells only, as the level in IFN-β-treated Huh-7 cells was within the ± 1.2 range set as background (Figure 1, panel B: miR-196b, miR-125a, miR-122a, miR-30c and miR-30b). Two miRs were modulated in an opposite manner in IFN-β-treated Huh-7 cells and in 21-5 replicon cells (Figure 1, panel C: miR-128b and miR-196a).

### Identification of common miRs modulated in different HCV replicon clones

**Figure 2:**
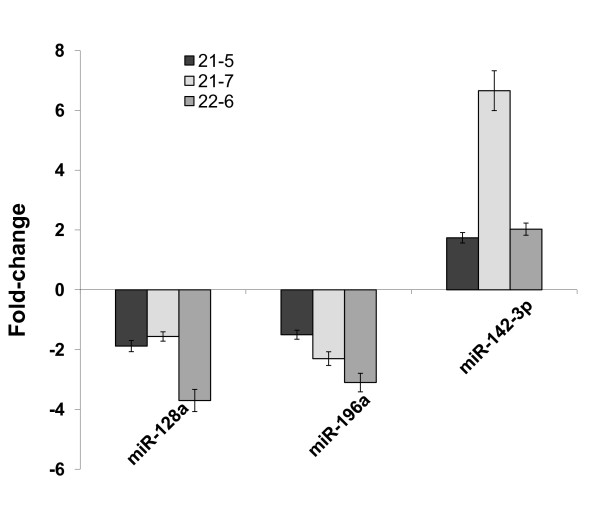
**Concordant expression of 3 miRs in different HCV replicon clones**. Total RNA from 21-5, 21-7 and 22-6 replicon cell lines was used to assess the level of miR-128a, miR-196a and miR-143-3p, using real time RT-PCR. miR levels from Huh-7 cells were set as the basis for the comparative results. The mean of three independent experiments ± SD is reported.

To exclude that the miR expression profile was peculiar of the 21-5 clone, we analyzed the expression level of the 16 miRs in two other HCV replicon clones, 22-6 and 21-7. The analysis revealed that 3 miRs showed concordant modulation in HCV clones as compared to Huh-7 cells (Figure 2). In particular, miR-128a and miR-196a were down-regulated while miR-142-3p was up-regulated in all HCV clones.

### Identification of candidate miR target genes

To predict target genes, which may be co-regulated by the 3 concordant miRs, we used miRGator, an on-line interface that uses multiple target prediction programs. It has been shown that, to date, no program is able to predict all experimentally confirmed target genes. Thus, to avoid as much as possible loss of putative target genes, relaxed options were used in miRGator [target genes predicted by at least one of the following programs: TargetScanS, miRanda and PicTar (4-way)]. After removal of multiple mRNAs corresponding to alternative mRNA transcripts from a single gene, individual gene lists were merged and a final list of 1981 total target genes was obtained, including genes controlled by at least one of the three miRs.

### Identification of genes common to miR target list and HCV microarray dataset

**Figure 3:**
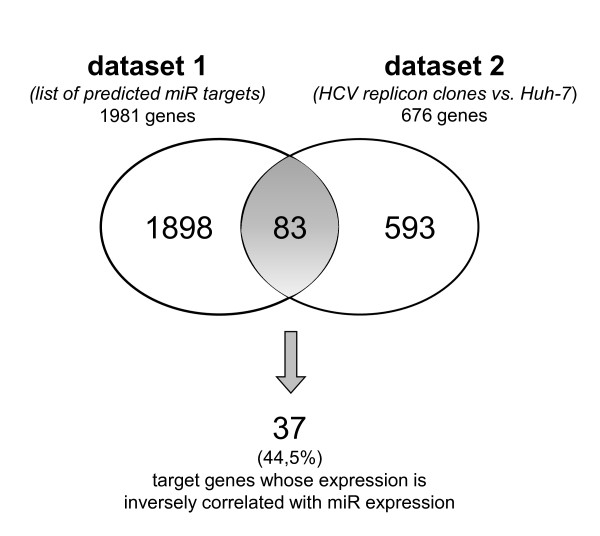
**Venn diagram showing validation of predicted target genes by microarray analysis**. Dataset 1 corresponds to the list of 1981 total target genes predicted for miR-128a, miR-196a and miR-142-3p; dataset 2 includes 676 genes differentially expressed in microarray analysis in all HCV clones as compared to Huh-7 cells. The number of predicted target genes differentially expressed in microarray analysis was 83, and 37 (44, 5%) showed an expression level inversely correlated with that of the corresponding miR.

The observation of an inverse relationship between levels of miRs and levels of their target mRNAs, due to mRNA degradation of target genes, provides opportunities for validation of predicted targets using microarray profiling [28]. On this basis, to determine the candidate target genes directly regulated by miR-128a, miR-196a and miR-142-3p, we overlapped two datasets: the list of 1981 total target genes predicted for the 3 miRs (dataset 1) and a microarray dataset including 676 genes (725 probes) modulated in all HCV clones as compared to Huh-7 cells (dataset 2) reported in our previous study [21]. As shown in Figure 3, 83 genes were common to both datasets indicating that target genes of the 3 miRs account for 12, 3% (83 out of 676) of the differentially expressed genes detected in all three HCV clones. The list of 83 genes, including relevant informations, is provided in Additional file 1, Table S1. As levels of most miRs and their target mRNAs exhibit an inverse expression relationship, we used gene expression profiling data to identify functional targets and validate target prediction, as previously reported [29]. By using this approach we found that 37 out of 83 (44, 5%) of the predicted target genes showed an expression level inversely correlated with that of the corresponding miR suggesting that, at least for these genes, a direct connection to miR regulation may be suggested. A complete list of the 37 genes (22 up-regulated and 15 down-regulated) and the corresponding 3 miRs is provided in Table 2. The list includes symbol, name, probe ID, fold-change (FC) and p-value of the genes all obtained from microarray dataset. In addition, a functional description of the genes by Panther Protein Classification System is enclosed [30]. Overlapping the 37 genes with the dataset of genes resulting from microarray analysis of Huh-7.5 cells infected with HCV genotype 2a chimeric virus J6/JFH [31] revealed 4 common genes (RND3, LTB4DH, RIOK3 and CHAF1B) which showed the same direction of regulation (i.e. up-regulated or down-regulated genes) in both HCV clones and J6/JFH microarray datasets supporting the biological relevance of these genes in HCV replicative cycle. Finally, to explore the involvement of the identified genes in HCV response to endogenous IFN, we also overlapped the list of 37 genes with the dataset of 1996 human genes annotated in the INTERFEROME database [32]. As shown in Table 2, four genes (HNMT, XPO1, PMPCB and HMGB1) were identified as Interferon Regulated Genes (IRGs).

**Table 2:** Reverse regulatory association of 3 differentially expressed miRNAs and their 37 predicted target genes in HCV replicon clones *vs*. Huh-7

	Gene_Symbol	Gene_name	ProbeID	Fold-change	p-value	Biological process and Molecular function
**Down-regulated miR**						
miR-128a	CAMTA1	calmodulin binding transcription activator 1	164871	1.426	0.045	Signal transduction/Calcium mediated signaling
miR-128a	CAST	calpastatin	222724	1.788	0.002	Cysteine protease inhibitor|Select regulatory molecule
miR-128a	CORO1C	coronin, actin binding protein, 1C	107201	1.402	0.027	Cytoskeletal protein|Actin binding cytoskeletal protein
miR-128a	PARK7	Parkinson disease (autosomal recessive, early onset) 7	136848	1.529	0.003	Nucleic acid binding
miR-128a	REPS1	RALBP1 associated Eps domain containing 1	147893	1.352	0.002	Intracellular protein traffic/Endocytosis
miR-128a	RND3^#^	Rho family GTPase 3	129829	2.161	0.041	G-protein|Small GTPase|Select regulatory molecule
miR-128a	SAA4	serum amyloid A4, constitutive	214434	1.880	0.029	Apolipoprotein;Defense/immunity protein
miR-128a	TMCC1	transmembrane and coiled-coil domain family 1	112169	1.369	0.031	unclassified
miR-128a	TRAPPC4	trafficking protein particle complex 4	205369	1.502	0.012	Membrane traffic protein
miR-128a	UBE2E3	ubiquitin-conjugating enzyme E2E 3 (UBC4/5 homolog, yeast)	201108	1.505	0.030	Ligase|Other ligase
miR-128a	VAMP8	vesicle-associated membrane protein 8 (endobrevin)	226943	2.586	0.011	SNARE protein|Membrane traffic protein
miR-196a	ARMET	arginine-rich, mutated in early stage tumors	144765	1.816	0.029	unclassified
miR-196a	HADH2	hydroxyacyl-Coenzyme A dehydrogenase, type II	142475	1.421	0.031	Oxidoreductase|Reductase|Dehydrogenase;Oxidoreductase
miR-196a	HIST1H2BD	histone 1, H2bd	148940	2.656	0.020	DNA binding
miR-196a	HNMT^▲^	histamine N-methyltransferase	227509	1.423	0.039	unclassified|IRG^▲^
miR-196a	LAMA4	laminin, alpha 4	101059	2.660	0.033	Extracellular matrix|Extracellular matrix linker protein
miR-196a	LTB4DH^#^	leukotriene B4 12-hydroxydehydrogenase	210882	1.961	0.001	Oxidoreductase|Reductase|Dehydrogenase;Oxidoreductase
miR-196a	PI3	peptidase inhibitor 3, skin-derived (SKALP)	128827	2.558	0.018	Proteolysis|Protein metabolism and modification
miR-196a	RIOK3^#^	RIO kinase 3 (yeast)	142169	3.226	0.018	Protein metabolism and modification|Protein phosphorylation
miR-196a	RRAGA	Ras-related GTP binding A	105497	1.243	0.023	G-protein|Small GTPase|Select regulatory molecule
miR-196a	ZMYND11	zinc finger, MYND domain containing 11	161540	1.665	0.036	Transcription factor|Transcription cofactor
miR-128a/miR-196a	CENPE^§^	centromere protein E, 312kDa	165425	1.436	0.018	Structural constituent of cytoskeleton
**Up-regulated miR**						
miR-142-3p	ZCCHC14	zinc finger, CCHC domain containing 14	200292	0.759	0.035	unclassified
miR-142-3p	XPO1^▲^	exportin 1 (CRM1 homolog, yeast)	105030	0.748	0.002	Intracellular protein transport|IRG^▲^
miR-142-3p	ATAD2	ATPase family, AAA domain containing 2	114584	0.745	0.007	Hydrolase activity
miR-142-3p	TRIM33	tripartite motif-containing 33	134526	0.714	0.004	Transcription cofactor;Nucleic acid binding|Transcription factor
miR-142-3p	RAB1A	RAB1A, member RAS oncogene family	122650	0.704	0.045	G-protein|Small GTPase|Select regulatory molecule
miR-142-3p	TFG	TRK-fused gene	210281	0.686	0.026	unclassified
miR-142-3p	PMPCB^▲^	peptidase (mitochondrial processing) beta	210046	0.684	0.015	Oxidoreductase activity|IRG^▲^
miR-142-3p	ZNF226	zinc finger protein 226	178824	0.682	0.043	Zinc finger transcription factor
miR-142-3p	ANKHD1	ankyrin repeat and KH domain containing 1	224628	0.658	0.006	unclassified
miR-142-3p	CXADR	coxsackie virus and adenovirus receptor	108284	0.620	0.025	Other receptor|Receptor
miR-142-3p	CHAF1B^#^	chromatin assembly factor 1, subunit B (p60)	174351	0.586	0.017	Nucleic acid binding|Chromatin/chromatin-binding protein
miR-142-3p	HMGB1^▲^	high-mobility group box 1	151074	0.586	0.011	Proinflammatory cytokine|IRG^▲^
miR-142-3p	BNIP3	BCL2/adenovirus E1B 19kDa interacting protein 3	109165	0.551	0.000	unclassified
miR-142-3p	COG4	component of oligomeric golgi complex 4	151150	0.394	0.042	Other RNA-binding protein|Nucleic acid binding
miR-142-3p	USH1C	Usher syndrome 1C (autosomal recessive, severe)	195953	0.367	0.016	Cytoskeletal protein

^§ ^Gene targeted by two down-regulated miRs

^▲ ^Interferon regulated gene (IRG) according to the INTERFEROME database

^# ^Gene showing same direction of regulation (up or down) in microarray analysis of HCV genotype 2a chimeric virus J6/JFH infection

### Biological functions of the miR target genes

To classify genes into biological categories, we analyzed the Gene Ontology annotations of the 37 common genes with the Panther Protein Classification System [30]. As shown in Table 3, Panther System found several functional categories that were significantly enriched in this gene set compared to the entire NCBI reference list of human genome. We considered, as potentially interesting, only categories showing a p-value ≤ 0.05, as determined by the binomial statistic [33]. The 37 genes of the dataset were significantly classified by the Panther system in 6 biological processes (i.e., processes in which genes participate) and 3 molecular functions (i.e., biological functions of gene products). Compared with the NCBI reference list of human genome, this dataset showed a larger proportion of genes encoding proteins involved in chromatin binding and architecture, organelle organization, intracellular transport and neurotransmitter secretion (Table 3). In addition, genes associated with catalytic activity, enzyme regulator activity and chromatin binding were represented much more abundantly in the dataset. Interestingly, genes involved in the Ubiquitin proteasome pathway were also present in the dataset (Table 3). Additional file 2, Table S2 reports the complete list of the genes that are responsible for statistical enrichment of each category shown in Table 3

**Table 3:** Panther classification of Biological processes, Molecular functions and Pathways significantly enriched in the set of 37 genes

	**Observed Genes**^**•**^	**Expected Genes**^**••**^	**p-value**^**•••**^
**BIOLOGICAL PROCESS**			
**Cellular process**			
Establishment or maintenance of chromatin architecture	4	0.55	2.12E-03
**Cellular component organization**			
Organelle organization	4	0.59	2.85E-03
**Transport**			
Intracellular protein transport	9	2.98	2.16E-03
Vesicle-mediated transport	7	2.10	4.26E-03
Endocytosis	4	1.04	1.96E-02
**System process**			
Neurotransmitter secretion	3	0.63	2.45E-02
			
**MOLECULAR FUNCTION**			
**Catalytic activity**			
GTPase activity	3	0.52	1.53E-02
**Enzyme regulator activity**			
Peptidase inhibitor activity	2	0.32	4.08E-02
**Binding**			
Chromatin binding	2	0.34	4.59E-02
			
**PATHWAYS**			
**Ubiquitin proteasome pathway**	2	0.13	7.19E-03

^• ^Number of genes, in the dataset of 37, that map to the indicated Panther classification categories

^•• ^Expected number of genes, in the dataset of 37, based on the NCBI reference list of human genome

^••• ^p-value as determined by the binomial statistic. A cutoff of 0.05 has been applied

## Discussion

In the present study we analyzed the effect of HCV replication on the expression of selected miRs involved in the IFN-pathway. In particular, we identified 3 miRs that are equally modulated by HCV in three HCV replicon clones and by IFN treatment. Moreover, we also identified 37 out of 83 predicted target genes, differentially expressed in HCV replicon cells, which are most likely functional targets of these 3 miRs: in fact they showed an inverse expression relationship with the level of the 3 miRs, as described for true targets. These genes could be implicated in regulation of the host response to HCV.

About one half predicted targets did not show the expected inverse expression relationship with miR level, but this result is not surprising. First, computational prediction by miRgator was based on predictions from the widely used programs miRanda, TargetScanS and PicTar (see Materials and Methods). To date, these programs, as well as any other available prediction program, still have a very high false-positive rate, estimated to be up to 40% [34,35]: i.e. up to 40% genes predicted to be targeted by a miR are not, actually, true targets, and they will not show any inverse relationship. Second, each mRNA is usually targeted by multiple miRs and, in addition, each mRNA has its own set of modulating miRs. Whether or not a change in the level of a single miR can prevail on the effect of the other miRs simultaneously targeting the same mRNA in physiological conditions is, at present, poorly understood. However, it is possible in some cases it will produce no effect and, thus, even a true target will not show the expected inverse relationship.

The recent discovery of the roles of miRs in many human diseases suggests that studies exploring the relationship between HCV and miRs/mRNA may provide new insights into host cell response to HCV infection. Importantly, this approach also gives the opportunity to identify viral mechanisms that control the antiviral defense. Recently, it has been demonstrated that five IFN-β-modulated miRs (miR-196, miR-296, miR-351, miR-431 and miR-448) showed significant effect on HCV replication and at least two of them (miR-196 and miR-448) are directly targeting the HCV genomic RNA [16]. Moreover, it seems that level of miR-122 is inversely correlated with the antiviral defense [16,36,37].

Our expression analysis revealed that miR-196a was down-regulated in all three HCV replicon clones. Thus, at least for this component of the pathway, it seems that modulation of IFN-miRs may be altered by HCV in replicon cells. Interestingly, this miR targets the HCV RNA [16], thus, down-regulation of miR-196a may indirectly influence viral replication also by up-regulation of specific target genes. Accordingly, 11 genes, controlled by miR-196a, showed by microarray analysis an inverse expression relationship suggesting that they can be likely considered functional targets of miR-196a. Moreover, gene ontology analysis of the 11 genes highlights that some of them are really involved in pathways such as, extracellular matrix constitution (LAM4), oxidative stress (HADH2, LTB4DH) and cytoskeletal network (CENPE), which are relevant for HCV RNA replication [7,38,39].

As for the IFN-β-regulated miR-296, miR-351, miR-431, miR-448 and miR-122 (16) our data indicate that their expression in different HCV replicon clones is either not concordant (miR-296, and miR-122a) or not detected (miR-351, miR-431 and miR-448). In particular, miR-122a was down-regulated in 21-5 and 21-7 clones but its level was not modified in clone 22-6 (data not shown) while miR-296 was down-regulated in 21-5 clone and up-regulated in clone 22-6 and 21-7, respectively (data not shown). Moreover, miR 351, miR-431 and miR-448 were not detected in all clones examined supporting, at least for miR-448, what found in human biopsies where miR-448 is totally absent [37].

Interestingly, we observed that two miRs, miR-142-3p and miR-128a, were up-regulated and down-regulated respectively by IFN-β treatment of Huh-7 cells and in HCV replicon-expressing cells. Although modulation of these 2 miRs by IFN-β has never been described before, it is consistently observed in all clones suggesting that it may be part of the endogenous IFN response to HCV. The IFN pathway is a highly regulated process and several controls has been evolved to activate and turn off this pathway. In this scenario, the temporal modulation of specific miRs seems to represent one of the control elements [40]. It is important to note that this process cannot properly occur in cells sustaining HCV replication. In this case a chronic up or down-regulation of IFN-miRs, likely induced by the virus, may negatively affect the control of the pathway finally improving the efficacy of the antiviral effectors. It would be interesting to investigate whether the experimental use of miR inhibitors (for miR-142-3p) or miR mimics (for miR-128a) could influence the control of the endogenous IFN system.

Among the 37 predicted target genes showing an inverse expression relationship with the 3 miRs, four genes were identified as Interferon Regulated Genes (IRGs) according to the INTERFEROME database [32]. One of these genes (HNMT) is a predicted target gene of miR-196a while the other three (XPO1, PMPCB and HMGB1) are all targeted by miR-142-3p. Importantly, in autoimmune diseases the high mobility group box 1 (HMGB1) protein was identified as a component of immune complex-containing DNA or RNA, which may act as endogenous IFN-β inducer [41]. Down-regulation of HMGB1 gene (fold change 0, 58; p-value 0, 01) in all HCV replicon clones suggests that it might contribute to impair the activation of the IFN signaling. Currently, the role of these four IRGs in the IFN response to HCV replication is unknown. Thus, unraveling their contribution to the regulation of the IFN response may reveal new mechanisms of viral persistence.

Gene Ontology annotations of the 37 common genes also revealed the presence of two genes, UBE2E3 and ATAD2 targets of miR-128a and miR-142-3p respectively, which are involved in the Ubiquitin proteasome pathway. The contribution of this pathway to HCV subversion of the IFN response has never been investigated. This is a quite interesting issue as several viruses use the ubiquitin proteasome system to destabilize proteins, such as IRF3 and STAT proteins, that are important for transcription of Interferon and Interferon-stimulated genes [42].

In attempt to validate our data, we found that 4 out of 37 genes, targeted by the 3 miRs, were also modulated, in a concerted fashion, in HCV genotype 2a chimeric virus J6/JFH microarray datasets [31] supporting the biological relevance of our results. In addition, 6 genes (4 up-regulated: CEACAM1, DDIT3, SCARB2, ZFAND2A; and 2 down-regulated: C9orf19 and RPL5) selected from the HCV clones microarray dataset were found to be modulated in a same way in liver biopsies of patients showing non-CC IL28B polymorphism [20]. This polymorphism is not a good predictor of response to IFN therapy and it is also associated with higher level of ISG expression in the liver and propension to chronicity. So, it can be speculated that modulation of IFN signature as mediated by common miRs in replicon cells, can mirror de-regulation of the IFN signaling proposed for such patients.

## Conclusions

In the present study we used the HCV replicon system to identify IFN-regulated miRs that are modulated by HCV RNA replication. By a combined approach, based on Real-Time PCR, bioinformatic prediction and microarray analysis, we identified 3 IFN-β-regulated miRs and 37 genes, which are likely their functional targets, commonly modulated by HCV in three replicon clones. Gene ontology classified the 37 genes into functional categories potentially implicated in the control of antiviral response by HCV infection. The future design of siRNAs directed against some of these genes and the use of miRs and antimiRs may provide an experimental background for the development of therapeutic strategies aimed at the recovering of protective innate responses in HCV infections.

## Methods

### Cell lines

The Huh-7 cells carrying the Sfl HCV full-length replicon (genotype 1b) were obtained from Dr. R. Bartenschlager. The 21-5, 21-7 and 22-6 clones are cell lines that stably replicates the HCV replicon and were passaged as described [43-45]. HCV replicon cells were cultured in complete DMEM supplemented with 10% FCS, antibiotics, 1× non-essential amino acids, and 250 μg/ml (21-5, 21-7) and 500 μg/ml (22-6) G418. Huh-7 cells were stimulated with 100 UI/ml IFN-β for 16 h.

### Quantitation of miRNAs

Total RNA was extracted from 1 × 10^6 ^cells using miRNeasy mini kit (QIAGEN) according to manufacturer's instructions and quantified by Bioanalyzer 2100 (Agilent Technologies). TaqMan^® ^MicroRNA Assays were used to quantitate miRs according to manifacturer's instructions (Applied Biosystems, Foster City, CA). A single TaqMan MicroRNA assay is used for each miR. All necessary primers and TaqMan probes are provided by the manufacturer with each assay, but details about sequence of primers and probes are not available (patented). Each TaqMan MicroRNA assay includes: (a) a "looped" primer, specific for each miR, for the reverse transcription step (carried out with the TaqMan^® ^MicroRNA Reverse Transcription Kit) and (b) a pair of "conventional" primers for amplification as well as a fluorescently labeled TaqMan probe for detection for the Real-Time amplification step (carried out with the TaqMan Universal PCR Master Mix).

In brief, 5 ng total RNA was reverse transcribed in 7.5 μl reaction volume containing 50 nM looped miR-specific primer, 1× RT buffer, 0.25 mM each dNTPs, 3.33 U/μl MultiScribe™ reverse transcriptase and 0.25 U/μl RNAse inhibitor. The reactions were incubated in an ABI Prism 7000 Sequence Detection System (Applied Biosystems) in a 96-well plate for 30 min at 16°C, 30 min at 42°C, followed by 5 min at 85°C, and then held at 4°C. Reverse transcription products were diluted three times with nuclease-free water prior to setting up PCR reactions. Each microRNA Real-Time PCR (10 μl volume) was carried out in triplicate, and each 10 μl reaction mixture included 2 μl of diluted reverse transcription reaction product, 5 μl of 2X TaqMan^® ^Universal PCR Master Mix, 1X assay mix (including TaqMan^® ^probe and forward and reverse primers). The reactions were incubated in an ABI Prism 7000 Sequence Detection System (Applied Biosystems) in 96-well plates at 95°C for 10 min, followed by 40 cycles of 95°C for 15 sec and 60°C for 1 min. Fold induction was calculated by 2^-ΔCt ^method [46] using the level of Huh-7 cell line as a calibrator.

### Prediction of genes targeted by modulated miRs

Putative gene targets of miRs found to be modulated in HCV clones were predicted by means of the miRGator program (available at http://genome.ewha.ac.kr/miRGator/miRNAexpression.html) that allows to combine gene predictions by TargetScanS, miRanda and PicTar softwares. To avoid loss of potential targets, a relaxed option was selected (retrieval of genes predicted by at least one program), so as to obtain for each miR a gene list as wide as possible.

### Gene network pathway analysis

Gene Ontology (GO) annotations were analyzed with the Panther Protein Classification System [30] to identify functional annotations that were significantly enriched in this gene set compared to the entire human genome. Gene lists modulated by HCV were mapped onto biological pathways that were significantly represented.

## Competing interests

The authors declare that they have no competing interests.

## Authors' contributions

RB had been involved in conception and design of the study, data analysis and interpretation and revised the paper critically; CM and PT carried out real-time PCR assays, most of microarray experiments and contributed to data interpretation; ET and ES carried out cell culture experiments; AC and UV participated in data analysis; AB contributed to the revision of the paper critically for important intellectual content; ARC had been involved in conception and design of the study, data analysis and interpretation and drafting the manuscript. All the authors had given final approval of the version to be published.

## Supplementary Material

Additional file 1 **Table S1**. List of 83 genes resulting from overlapping (a) predicted target genes of miR-128a or miR-196a or miR-143-3p and (b) genes experimentally showing an expression change in HCV replicon clones vs. parental Hu-h7 cells by microarray analysis [21]. Click here for file

Additional file 2 **Table S2**. Individual genes in each of the classification groups found to be significantly enriched in the set of 37 genes by Panther. Click here for file

## References

[B1] Hoofnagle JH Hepatitis C: the clinical spectrum of disease Hepatology 1997 26 15S 20S 10.1002/hep.510260703 9305658

[B2] Lauer GM Walker BD Hepatitis C virus infection N Engl J Med 2001 345 41 52 Review 10.1056/NEJM200107053450107 11439948

[B3] Walters KA Syder AJ Lederer SL Diamond DL Paeper B Rice CM Katze MG Genomic analysis reveals a potential role for cell cycle perturbation in HCV-mediated apoptosis of cultured hepatocytes PLoS Pathog 2009 5 e1000269 10.1371/journal.ppat.1000269 19148281 PMC2613535

[B4] Walters KA Joyce MA Thompson JC Smith MW Yeh MM Proll S Zhu LF Gao TJ Kneteman NM Tyrrell DL Katze MG Host-specific response to HCV infection in the chimeric SCID-beige/Alb-uPA mouse model: role of the innate antiviral immune response PLoS Pathog 2006 2 e59 10.1371/journal.ppat.0020059 16789836 PMC1480599

[B5] Walters KA Smith MW Pal S Thompson JC Thomas MJ Yeh MM Thomas DL Fitzgibbon M Proll S Fausto N Gretch DR Carithers RL Jr Shuhart MC Katze MG Identification of a specific gene expression pattern associated with HCV-induced pathogenesis in HCV- and HCV/HIV-infected individuals Virology 2006 350 453 64 10.1016/j.virol.2006.02.030 16574185

[B6] Smith MW Walters KA Korth MJ Fitzgibbon M Proll S Thompson JC Yeh MM Shuhart MC Furlong JC Cox PP Thomas DL Phillips JD Kushner JP Fausto N Carithers RL Jr Katze MG Gene expression patterns that correlate with hepatitis C and early progression to fibrosis in liver transplant recipients Gastroenterology 2006 130 179 87 10.1053/j.gastro.2005.08.015 16401481

[B7] Smith MW Yue ZN Korth MJ Do HA Boix L Fausto N Bruix J Carithers RL Jr Katze MG Hepatitis C virus and liver disease: global transcriptional profiling and identification of potential markers Hepatology 2003 38 1458 67 14647057 10.1016/j.hep.2003.09.024

[B8] Bigger CB Guerra B Brasky KM Hubbard G Beard MR Luxon BA Lemon SM Lanford RE Intrahepatic gene expression during chronic hepatitis C virus infection in chimpanzees J Virol 2004 78 13779 92 10.1128/JVI.78.24.13779-13792.2004 15564486 PMC533929

[B9] Lanford RE Guerra B Bigger CB Lee H Chavez D Brasky KM Lack of response to exogenous interferon-alpha in the liver of chimpanzees chronically infected with hepatitis C virus Hepatology 2007 46 999 1008 17668868 10.1002/hep.21776 PMC2386986

[B10] Lanford RE Guerra B Lee H Chavez D Brasky KM Bigger CB Genomic response to interferon-alpha in chimpanzees: implications of rapid downregulation for hepatitis C kinetics Hepatology 2006 43 961 72 10.1002/hep.21167 16628626

[B11] Su AI Pezacki JP Wodicka L Brideau AD Supekova L Thimme R Wieland S Bukh J Purcell RH Schultz PG Chisari FV Genomic analysis of the host response to hepatitis C virus infection Proc Natl Acad Sci USA 2002 99 15669 74 10.1073/pnas.202608199 12441396 PMC137774

[B12] Bièche I Asselah T Laurendeau I Vidaud D Degot C Paradis V Bedossa P Valla DC Marcellin P Vidaud M Molecular profiling of early stage liver fibrosis in patients with chronic hepatitis C virus infection Virology 2005 332 130 44 10.1016/j.virol.2004.11.009 15661146

[B13] Lau DT Fish PM Sinha M Owen DM Lemon SM Gale M Jr Interferon regulatory factor-3 activation, hepatic interferon-stimulated gene expression, and immune cell infiltration in hepatitis C virus patients Hepatology 2008 47 799 809 10.1002/hep.22076 18203148

[B14] Lau DT Luxon BA Xiao SY Beard MR Lemon SM Intrahepatic gene expression profiles and alpha-smooth muscle actin patterns in hepatitis C virus induced fibrosis Hepatology 2005 42 273 81 10.1002/hep.20767 15986378

[B15] Helbig KJ Lau DT Semendric L Harley HA Beard MR Analysis of ISG expression in chronic hepatitis C identifies viperin as a potential antiviral effector Hepatology 2005 42 702 10 10.1002/hep.20844 16108059

[B16] Pedersen IM Cheng G Wieland S Volinia S Croce CM Chisari FV David M Interferon modulation of cellular microRNAs as an antiviral mechanism Nature 2007 449 919 22 10.1038/nature06205 17943132 PMC2748825

[B17] Gale M Jr Foy EM Evasion of intracellular host defence by hepatitis C virus Nature 2005 436 939 45 Review. Erratum in: Nature. 2005 Sep 8;437(7056):290 10.1038/nature04078 16107833

[B18] Ge D Fellay J Thompson AJ Simon JS Shianna KV Urban TJ Heinzen EL Qiu P Bertelsen AH Muir AJ Sulkowski M McHutchison JG Goldstein DB Genetic variation in IL28B predicts hepatitis C treatment-induced viral clearance Nature 2009 461 399 401 10.1038/nature08309 19684573

[B19] Thomas DL Thio CL Martin MP Qi Y Ge D O'Huigin C Kidd J Kidd K Khakoo SI Alexander G Goedert JJ Kirk GD Donfield SM Rosen HR Tobler LH Busch MP McHutchison JG Goldstein DB Carrington M Genetic variation in IL28B and spontaneous clearance of hepatitis C virus Nature 2009 461 798 801 10.1038/nature08463 19759533 PMC3172006

[B20] Urban TJ Thompson AJ Bradrick SS Fellay J Schuppan D Cronin KD Hong L McKenzie A Patel K Shianna KV McHutchison JG Goldstein DB Afdhal N IL28B genotype is associated with differential expression of intrahepatic interferon-stimulated genes in patients with chronic hepatitis C Hepatology 2010 52 1888 96 10.1002/hep.23912 20931559 PMC3653303

[B21] Ciccaglione AR Marcantonio C Tritarelli E Ferraris A Tataseo P Bruni R Dallapiccola B Gerosolimo G Costantino A Rapicetta M Microarray analysis identifies a common set of cellular genes modulated by different HCV replicon clones BMC Genomics 2008 9 309 10.1186/1471-2164-9-309 18590516 PMC2474623

[B22] Wakita T Pietschmann T Kato T Date T Miyamoto M Zhao Z Murthy K Habermann A Krausslich HG Mizokami M Bartenschlager R Liang TJ Production of infectious hepatitis C virus in tissue culture from a cloned viral genome Nat Med 2005 11 791 796 10.1038/nm1268 15951748 PMC2918402

[B23] Zhong J Gastaminza P Cheng G Kapadia S Kato T Burton DR Wieland SF Uprichard SL Wakita T Chisari FV Robust hepatitis C virus infection in vitro Proc Natl Acad Sci USA 2005 102 9294 9299 10.1073/pnas.0503596102 15939869 PMC1166622

[B24] Lindenbach BD Evans MJ Syder AJ Wölk B Tellinghuisen TL Liu CC Maruyama T Burton DR McKeating JA Rice CM Complete replication of hepatitis C virus in cell culture Science 2005 309 623 6 10.1126/science.1114016 15947137

[B25] Jopling CL Yi M Lancaster AM Lemon SM Sarnow P Modulation of hepatitis C virus RNA abundance by a liver-specific microRNA Science 2005 309 1577 1581 10.1126/science.1113329 16141076

[B26] O'Connell RM Taganov KD Boldin MP Cheng G Baltimore D MicroRNA-155 is induced during the macrophage inflammatory response Proc Natl Acad Sci USA 2007 104 1604 9 10.1073/pnas.0610731104 17242365 PMC1780072

[B27] Taganov KD Boldin MP Chang KJ Baltimore D NF-kappaB-dependent induction of microRNA miR-146, an inhibitor targeted to signaling proteins of innate immune responses Proc Natl Acad Sci USA 2006 103 12481 6 10.1073/pnas.0605298103 16885212 PMC1567904

[B28] Peng X Li Y Walters KA Rosenzweig ER Lederer SL Aicher LD Proll S Katze MG Computational identification of hepatitis C virus associated microRNA-mRNA regulatory modules in human livers BMC Genomic 2009 10 373 10.1186/1471-2164-10-373 PMC2907698 19671175

[B29] Huang JC Babak T Corson TW Chua G Khan S Gallie BL Hughes TR Blencowe BJ Frey BJ Morris QD Using expression profiling data to identify human microRNA targets Nat Methods 2007 4 1045 1049 10.1038/nmeth1130 18026111

[B30] Mi H Lazareva-Ulitsky B Loo R Kejariwal A Vandergriff J Rabkin S Guo N Muruganujan A Doremieux O Campbell MJ Kitano H Thomas PD The PANTHER database of protein families, subfamilies, functions and pathways Nucleic Acids Res 2005 33 D284 8 10.1093/nar/gki418 15608197 PMC540032

[B31] Walters KA Syder AJ Lederer SL Diamond DL Paeper B Rice CM Katze MG Genomic analysis reveals a potential role for cell cycle perturbation in HCV-mediated apoptosis of cultured hepatocytes PLoS Pathog 2009 5 e1000269 10.1371/journal.ppat.1000269 19148281 PMC2613535

[B32] Samarajiwa SA Forster S Auchettl K Hertzog PG INTERFEROME: the database of interferon regulated genes Nucleic Acids Research 2009 37 Database D852 7 10.1093/nar/gkn732 PMC2686605 18996892

[B33] Cho RJ Campbell MJ Transcription, genomes, function Trends Genet 2000 16 409 415 Review 10.1016/S0168-9525(00)02065-5 10973070

[B34] Sethupathy P Megraw M Hatzigeorgiou AG A guide through present computational approaches for the identification of mammalian microRNA targets Nat Methods 2006 3 881 886 10.1038/nmeth954 17060911

[B35] Martin G Schouest K Kovvuru P Spillane C Prediction and validation of microRNA targets in animal genomes J Biosci 2007 32 6 1049 1052 17954966 10.1007/s12038-007-0106-0

[B36] Sarasin-Filipowicz M Oakeley EJ Duong FH Christen V Terracciano L Filipowicz W Heim MH Interferon signaling and treatment outcome in chronic hepatitis C Proc Natl Acad Sci USA 2008 105 7034 9 10.1073/pnas.0707882105 18467494 PMC2383932

[B37] Sarasin-Filipowicz M Krol J Markiewicz I Heim MH Filipowicz W Decreased levels of microRNA miR-122 in individuals with hepatitis C responding poorly to interferon therapy Nat Med 2009 15 31 3 10.1038/nm.1902 19122656

[B38] Bost AG Venable D Liu L Heinz BA Cytoskeletal requirements for hepatitis C virus (HCV) RNA synthesis in the HCV replicon cell culture system J Virol 2003 77 4401 4408 10.1128/JVI.77.7.4401-4408.2003 12634397 PMC150619

[B39] Korenaga M Wang T Li Y Showalter LA Chan T Sun J Weinman SA Hepatitis C virus core protein inhibits mitochondrial electron transport and increases reactive oxygen species (ROS) production J Biol Chem 2005 280 37481 37488 10.1074/jbc.M506412200 16150732

[B40] Witwer KW Sisk JM Gama L Clements JE MicroRNA regulation of IFN-beta protein expression: rapid and sensitive modulation of the innate immune response J Immunol 2010 184 2369 76 10.4049/jimmunol.0902712 20130213 PMC3076721

[B41] Finke D Eloranta ML Rönnblom L Endogenous type I interferon inducers in autoimmune diseases Autoimmunity 2009 42 349 52 Review 10.1080/08916930902831829 19811298

[B42] McInerney GM Karlsson Hedestam GB Direct cleavage, proteasomal degradation and sequestration: three mechanisms of viral subversion of type I interferon responses J Innate Immun 2009 1 599 606 Review 10.1159/000235861 20375615

[B43] Frese M Schwarzle V Barth K Krieger N Lohmann V Mihm S Haller O Bartenschlager R Interferon-gamma inhibits replication of subgenomic and genomic hepatitis C virus RNAs Hepatology 2002 35 694 703 10.1053/jhep.2002.31770 11870386

[B44] Pietschmann T Lohmann V Kaul A Krieger N Rinck G Rutter G Strand D Bartenschlager R Persistent and transient replication of full-length hepatitis C virus genomes in cell culture J Virol 2002 76 4008 4021 10.1128/JVI.76.8.4008-4021.2002 11907240 PMC136109

[B45] Ciccaglione AR Stellacci E Marcantonio C Muto V Equestre M Marsili G Rapicetta M Battistini A Repression of interferon regulatory factor 1 by hepatitis C virus core protein results in inhibition of antiviral and immunomodulatory genes J Virol 2007 81 202 214 10.1128/JVI.01011-06 17050603 PMC1797261

[B46] Schmittgen TD Livak KJ Analyzing real-time PCR data by the comparative C(T) method Nat Protoc 2008 3 1101 1108 10.1038/nprot.2008.73 18546601

